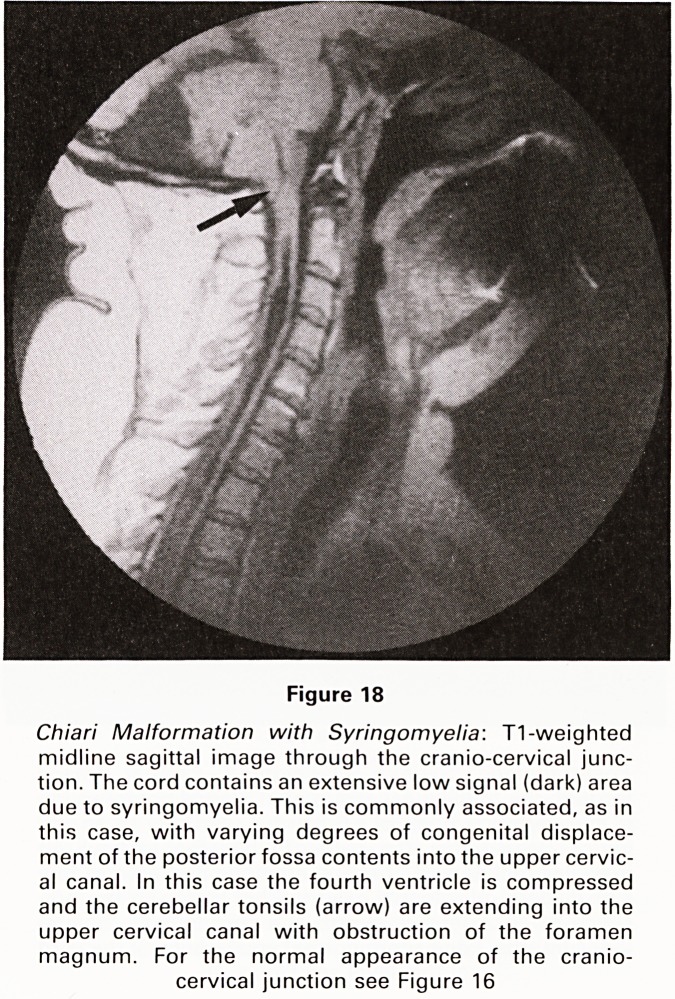# Magnetic Resonance Imaging of the CNS

**Published:** 1988-05

**Authors:** John R. Bradshaw, Timothy T. Lewis


### Magnetic Resonance Imaging of the CNS

**John R. Bradshaw FRCR** **Timothy T. Lewis FRCR**

In many respects the clinical development of MRI has mirrored that of Computed Tomography and it was in the brain that the new technique found its earliest and most successful application. Early limitations due to long data acquisition times were quickly overcome and the advent of simultaneous multiple slice techniques, higher field strengths, surface coils and other developments also helped to establish MRI in the forefront of CNS diagnosis. Considerations of cost aside, it is now generally accepted that the anatomical and pathological information currently achievable with MRI surpasses that possible with CT or myelography. This degree of effectiveness is achieved without ionising radiation or any invasive procedure.

Routine studies of the brain are readily produced in the three orthogonal planes: axial, coronal and sagittal. Oblique sections can also be produced. High resolution studies of specific areas such as the temporal bones or orbits can be achieved by specifically designed receiver coils applied directly to the surface. Similar coils are routinely used in studies of the spine. Practical limitations are the same as for any MRI examination and include problems with ferromagnetic materials, pacemakers etc. Since each examination may take up to an hour or more, a high degree of patient co-operation is desirable. The physical constraints within the scanning area render this imaging technique less suitable for patients under anaesthesia, on life support systems, or who are severely injured.

The full potential of MRI has yet to be realized but at present its ability to provide detailed anatomical studies together with tissue information in the form of T1 and T2 relaxation, flow effects etc. can usually enable a satisfactory diagnosis to be reached. A careful choice of appropriate imaging planes and sequences must be made for the clinical problem in hand. T1 weighted sequences provide some important signal information but their principal value lies in good anatomical resolution. While this has a significant bearing on diagnosis it is also of vital importance to the surgeon in planning surgical approach and feasibility of resection. T2 weighted sequences provide poorer resolution but are particularly sensitive to areas of abnormal tissue. Useful flow effects can be seen on various images and new specialized sequences are also under development. The advent of intra-venous paramagnetic contrast agents, soon to be available for clinical use, will provide further options of sequence choice and information on the blood brain barrier.

### BRAIN

*White matter disease*: At a very early stage in the clinical evaluation of MRI it became obvious that this technique was particularly sensitive to changes in white matter. MRI will show changes in multiple sclerosis (MS) at a very early stage but unfortunately the changes seen are non-specific. In older patients areas of deep ischaemia are a common finding and are often indistinguishable from the changes of MS. Other white matter diseases such as leucoencephalopathies are also elegantly defined by MRI.

*Congenital*: Because of its multiplanar capacity and exquisite delineation of anatomy, MRI is particularly suited to the accurate assessment of all congenital abnormalities in the head and cranio-cervical junction.

*Tumours*: Tumour tissue usually exhibits a prolongation of T1 and T2 relaxation times and is consequently particularly well visualized. Extra-axial lesions close to the skull base may be obscured by bone artefacts on CT but are seen unobscured on MRI. This absence of bone artefact also enables MRI to demonstrate small intracanalicular acoustic neuromas. Tumours arising within the cerebral substance, notably the glioma series, are well shown although the boundary between tumour tissue and reactive oedema may be difficult to define even with gadolinium enhancement.

*Infarction*: MRI is very sensitive for the detection of ischaemic changes. In fact such lesions are seen in a large proportion of the normal elderly population. While MRI may enlarge our understanding of the impact of ischaemia on the brain, its current role has little to offer that is not already available with CT. Infarcts in the brain stem and lower posterior fossa are much more readily identified on MRI, however.

*Haemorrhage*: Blood within brain tissue presents a complex variety of appearances on different MRI pulse sequences. This is due to the changing chemistry of the breakdown products of haemoglobin with time. While MRI studies can help to elucidate certain diagnostic problems where CT has encountered a haemorrhage in an undiagnostic form, the application of MR in haemorrhage is limited.

*Infection*: Because of its ability to detect early white matter changes it is now fairly certain that MRI can demonstrate encephalitis and cerebral abscesses before these entities are evident on other imaging techniques.

*Trauma*: MRI has the capacity to demonstrate all of the intracranial consequences of head injury including the earliest manifestations of oedema, but the severely injured or unconscious patient may present considerable problems in the vicinity of the magnet where life support systems are involved. The visualization of haemorrhage in the acute stage appears less reliable than by CT, and it seems unlikely that MRI will be widely used in the emergency management of trauma. Its capacity to delineate residual changes however, should be of considerable value.

*Pituitary*: It is now widely accepted that MRI will be the only imaging technique required in the assessment of pituitary tumours. Microadenomas are probably better visualized than on CT and the larger lesions are superbly delineated with full visualization of the relationships with adjacent blood vessels and the optic chiasm. These latter features are not well seen on CT.

*Posterior fossa*: CT scanning in the posterior fossa has always been limited by bone artefacts from the petrous bones and the skull base. The MRI imaging system is not susceptible to such artefacts and the contents of the posterior fossa and cranio-cervical junction are superbly demonstrated. Complete delineation of the brain stem, cerebellum, tentorium etc. can e routinely achieved, and it is now clear that where a patient's mode of presentation suggests pathology in the posterior fossa, then MRI should be the first and usually the only imaging technique required.

### SPINE

Soon after the first clinical MR images were produced, specially designed surface coils were introduced that made it possible to acquire useful studies of the spine. The technology evolved rapidly and MRI is now replacing CT and myelography for many of the disorders of the spinal cord and its adjacent structures. CT is very useful for the demonstration of bone disorders of the spine such as fractures, subluxation, spinal stenosis and lumbar disc protrusions. It suffers however, from bone artefacts which render it practically useless for the delineation of the spinal cord. Myelography is invasive and not without its side effects and dangers, but does provide good visualization of the cord and nerve roots. Its current advantage over MRI lies in its superior assessment of cervical disc brachalgia, but in all other respects; in cervical myelopathy, congenital lesions, dorsal spine disorders etc. MRI has clear advantages.

*Cord lesions*: It is for lesions within the cord such as tumours, syrinxes, myelomalacia etc. that MRI has been such an advance. MRI will demonstrate the extent and usually, the nature of, intramedullary lesions more accurately than any other technique and gives this information without the need for intrathecal contrast. It will usually be the only form of imaging necessary in such patients.

*Extra-axial lesions*: Neoplastic masses of both primary or secondary type together with inflammatory lesions of intradural or extradural location are well demonstrated. Myelography can now be avoided for these lesions. Pathology located in the dorsal area was often a problem on myelography and most can now be adequately resolved with MRI.

*Disc diseases*: Degenerative changes within the intervertebral disc, particularly those consequent on the loss of water in the nucleus pulposus, can be consistently demonstrated with MRI. In addition, cervical and dorsal disc disease producing significant myelopathy by cord compression are well shown by MRI. Disc protrusions associated principally with root compression, i.e. brachalgia or sciatica, are probably better assessed by CT or myelography, not least because of the number of patients and costs involved.

*Congenital*: The multiplanar capacity of MRI, together with accurate anatomical resolution and tissue characterization makes it the ideal imaging modality for congenital lesions of the spine. Meningomyeloceles, lipomas, cord tethering and displacements etc. can all be analysed in complete detail by this technique.

**Acknowledgements**

This paper could not have been compiled without the generous help and co-operation of the John James Bristol Foundation and the Trustees and staff of the Bristol MRI Centre. Our thanks are also due to Mrs M. Bradshaw and Miss S. Alden for typing the manuscript.

### SUGGESTED FURTHER READING

BRANT-ZAWADZKI, M. (1988) MR Imaging of the Brain. *Radiology* **166**, 1–10.

NORMAN, D. "The Spine". In "Magnetic Resonance Imaging of the Central Nervous System". Michael Brant-Zawadski (ed). Raven Press, New York, 1987.

**Figure 1**

*Multiple Sclerosis*: T2-weighted (spin-echo) axial image through the upper hemispheres. Numerous high signal (white) areas are shown due to demyelinating plaques. In a young patient such changes are typical of this disease. In older subjects however, the appearances can be difficult to distinguish from areas of deep ischaemia (see Figure 4). It is often not possible to discern whether the areas of demyelination are old or recent. This distinction may be possible with gadolinium enhancement. Plaques of demyelination can also be demonstrated in the cord

**Figure 2**

*Aqueduct Stenosis due to AVM*: T1-weighted (spin-echo) midline sagittal image. The brain-stem is clearly seen. The third and lateral ventricles are markedly dilated (arrows). The aqueduct is blocked and distorted by an ill-defined lesion (white arrowhead). The fourth ventricle is smaller than normal. For the normal appearance of the aqueduct in this plane see Figure 8. At surgery this was found to be a thrombosed arteriovenous malformation (AVM)

**Figure 3**

*Glioma of Left Temporal Lobe*: T1-weighted (inversion recovery) coronal image through the mid-temporal lobes. The distortion of the third and lateral ventricles is clearly shown. An ill-defined low signal (dark) area is seen occupying most of the left temporal lobe with upward displacement of the left sylvian fissure and invasion of the basal nuclei (arrow). Compare this with the normal anatomical configuration on the opposite side

**Figure 4**

*Deep Cerebral Ischaemia*: T2-weighted (spin-echo) axial image through the lateral ventricles. Patchy, but fairly symmetrical high signal (white) areas are present in the white matter around the ventricles. This is a common finding in older patients and is almost certainly the result of small vessel disease producing patches of deep cerebral ischaemia. The thin serpiginous black shadows lateral to the white areas are the branches of the middle cerebral arteries

**Figure 5**

*Infarction of the Cerebellum*: T2-weighted (Spin Echo) axial image through the lower cerebellum and brain stem. The anterior part of this image shows the nasal cavity in the midline with low signal (dark) paranasal sinuses on either side. Posteriorly the two cerebellar hemispheres can be distinguished with the brain-stem (arrow) lying almost between them on their antero-medial aspects. The right cerebellar hemisphere shows higher signal than the left due to extensive infarction in the territory of the right posterior inferior cerebellar artery (PICA). Unlike a tumour this is not causing any significant displacement of adjacent structures

**Figure 6**

*Thrombosed Aneurysm of Basilar Artery*: T1-weighted (inversion recovery) axial image through the brain-stem and cerebellum. The posterior half of the image is similar to that in Figure 5 but the most posterior structures visible are the tips of the occipital lobes (two arrows). Anterior to these can be seen the quite different texture of the cerebellar hemispheres. A rounded high signal area (white) represents flowing blood in the basilar artery (white arrowhead). Behind this is a rounded area of mixed signal due to a thrombosed giant aneurysm of the basilar artery. This is causing severe compression and displacement of the brain stem and fourth ventricle (large single arrow)

**Figure 7**

*Pituitary Microadenoma*: T1-weighted (spin-echo) sagittal image through pituitary fossa and brain-stem. The pituitary fossa is not enlarged but the gland does contain a small well-defined area of low signal (dark). This is due to a prolactin-secreting microadenoma which in this case appears to be cystic

**Figure 8**

*Pituitary Tumour*: T1-weighted (spin-echo) sagittal magnified image through the pituitary gland and brain-stem. The pituitary fossa is considerably expanded by a homogeneous tumour. The normal gland remnant (P) is flattened and lies below the mass. The pituitary stalk is clearly shown (S). Note also the aqueduct (A), fourth ventricle (F) and genu of corpus callosum (G). Coronal images delineate the relationship of such masses to the cavernous sinuses and optic chiasm

**Figure 9**

*Glioma of Optic Chiasm*: T2-weighted (spin-echo) magnified axial image through the supra-sellar area. Moving blood within the vessels of the Circle of Willis causes these structures to be clearly visible on most MRI sequences. The curved black structures seen here represent the middle cerebral (M), internal carotid (I), posterior communicating (P) and posterior cerebral (PC) arteries. Their relationship to the high signal tumour (white) is elegantly demonstrated

**Figure 10**

*Medulloblastoma*: T1-weighted (spin-echo) sagittal image of the midline. A large low signal mass is present in the lower cerebellum. This lesion is protruding into the displaced fourth ventricle (arrow) and causing distortion of the brain-stem. The third and lateral ventricles are also dilated. The vermis is a characteristic location for this tumour. Most are found in young patients, as in this case

**Figure 11**

*Acoustic Neuroma*: T1-weighted (inversion recovery) coronal image through the brain stem and cerebellum. The occipital horns of the lateral ventricles are dilated and the position of the tentorium between the inferior aspect of the occipital lobes and cerebellar hemispheres is well demonstrated. The brain stem is seen to be indented on its right side (arrow) by a low signal (dark) area. This, and the hydrocephalus, is the consequence of an acoustic neuroma

**Figure 12**

*Astrocytoma of Cervical Cord*: T2-weighted (spin echo) sagittal image through the cervical spine. Note the individual cervical vertebrae and the extensive area of mixed high and low signal. This is an astrocytoma expanding the cervical cord. The abnormal signal extends to C7 and up into the brain stem. This degree of delineation of a cord tumour is practically impossible by any other technique

**Figure 13**

*Post-traumatic Syrinx*: T1-weighted sagittal image through the midline cervical region. Partial compression of the vertebral body of C5 can be seen due to an old injury. Behind this, the cord can be seen to have an irregular outline and contains an elongated area of low signal (dark) of variable calibre. This is due to the presence of an extensive syrinx, the consequence of previous trauma. For a more normal appearance of the cord at this level see Figure 16

**Figure 14**

*Neurofibroma at C2*: T1-weighted midline sagittal image of cranio-cervical junction. The brainstem, cerebellum and upper cervical cord are clearly identified. The cord is markedly thinned and displaced posteriorly by a well defined mass anteriorly at the C2 level. The extramedullary nature of this lesion is obvious and the diagnosis rests between a meningioma or neurofibroma in this patient with neurofibromatosis

**Figure 15**

*Lymphoma in the Spinal Canal*: T1-weighted midline sagittal image in the dorsal region. The mid-dorsal vertebrae are readily identified. The dark shadows anterior to the spine represent flowing blood in the aorta and cardiac chambers. Behind the vertebrae can be seen two parallel shadows of almost equal thickness. The more anterior of these (closest to the vertebral bodies) is the mildly compressed dorsal cord. The posterior shadow (arrow) is an extradural collection of lymphoma tissue

**Figure 17**

*Dorsal Disc Protrusion*: T2-weighted midline sagittal image in the mid-dorsal area. On this sequence CSF is shown white and the cord a darker filling defect within it. Normal discs have a high signal and two of this patient's discs are degenerate with a low signal (dark) (arrows). The lower of these has protruded extensively into the spinal canal and shows unusually low signal due to calcification

**Figure 16**

*Cervical disc protrusion*: T1-weighted midline sagittal image through the cervical spine. The individual cervical vertebrae and the intervening discs are well demonstrated and the airways are shown anteriorly. The normal cervical cord is seen throughout its length but is indented anteriorly by a disc protrusion at C5/6 (arrow)

**Figure 18**

*Chiari Malformation with Syringomyelia*: T1-weighted midline sagittal image through the cranio-cervical junction. The cord contains an extensive low signal (dark) area due to syringomyelia. This is commonly associated, as in this case, with varying degrees of congenital displacement of the posterior fossa contents into the upper cervical canal. In this case the fourth ventricle is compressed and the cerebellar tonsils (arrow) are extending into the upper cervical canal with obstruction of the foramen magnum. For the normal appearance of the cranio-cervical junction see Figure 16

## Figures and Tables

**Figure 1 f1:**
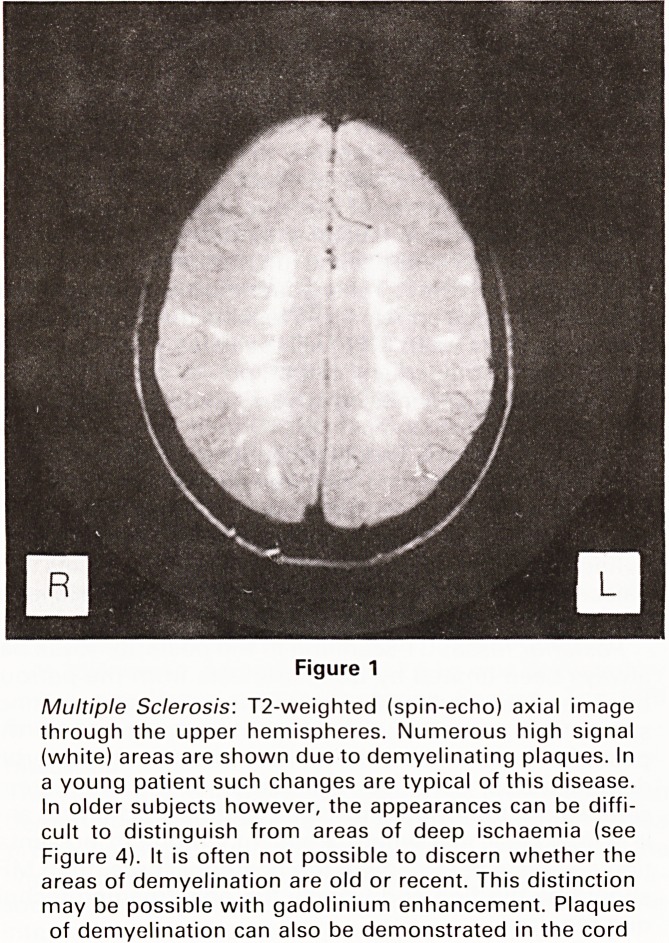


**Figure 2 f2:**
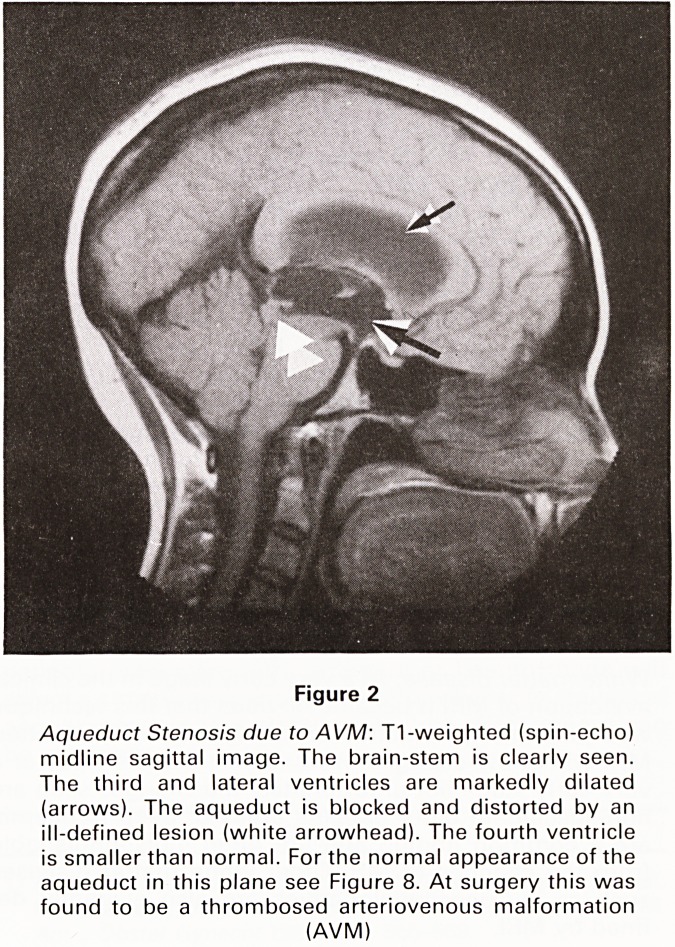


**Figure 3 f3:**
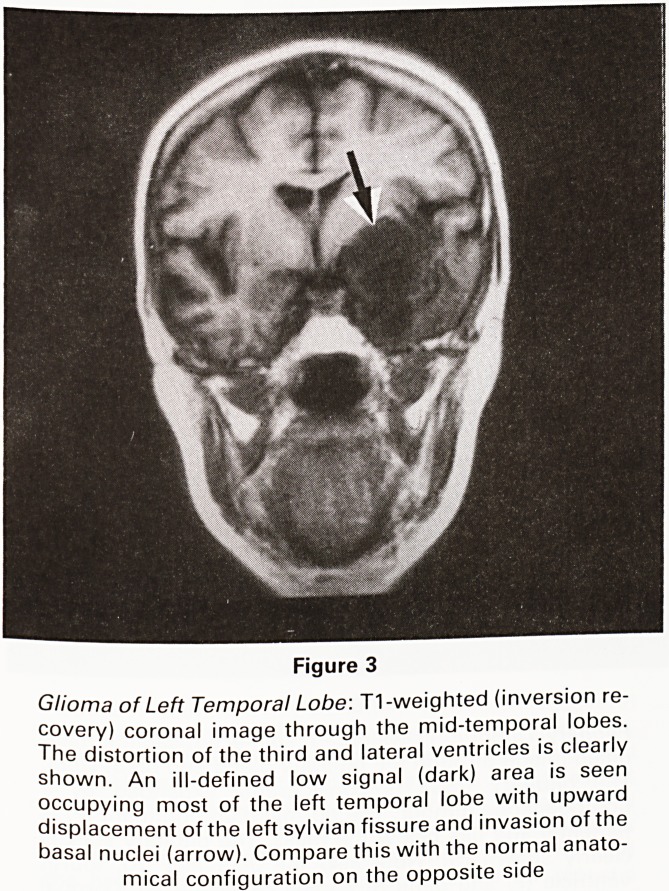


**Figure 4 f4:**
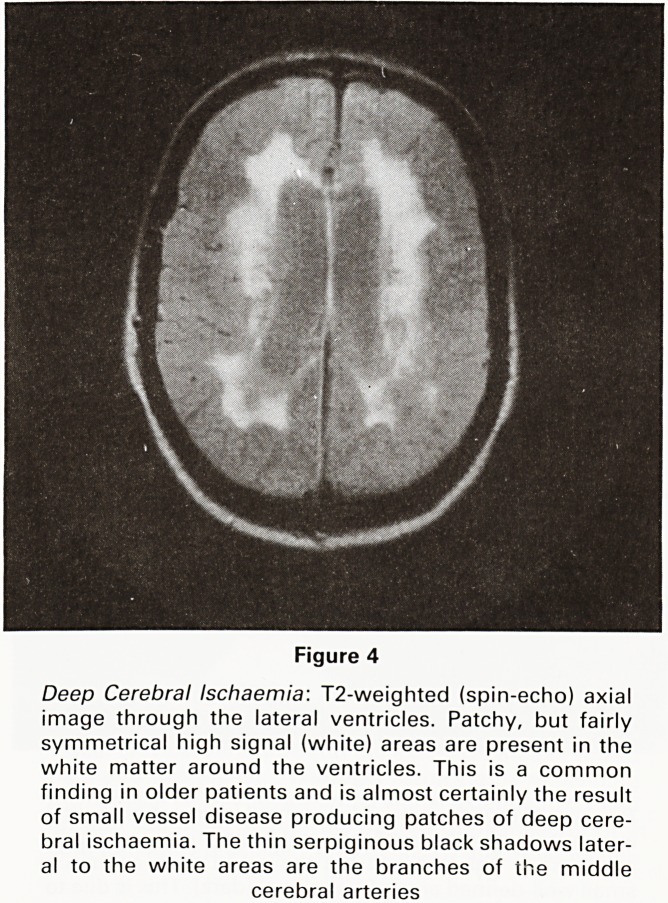


**Figure 5 f5:**
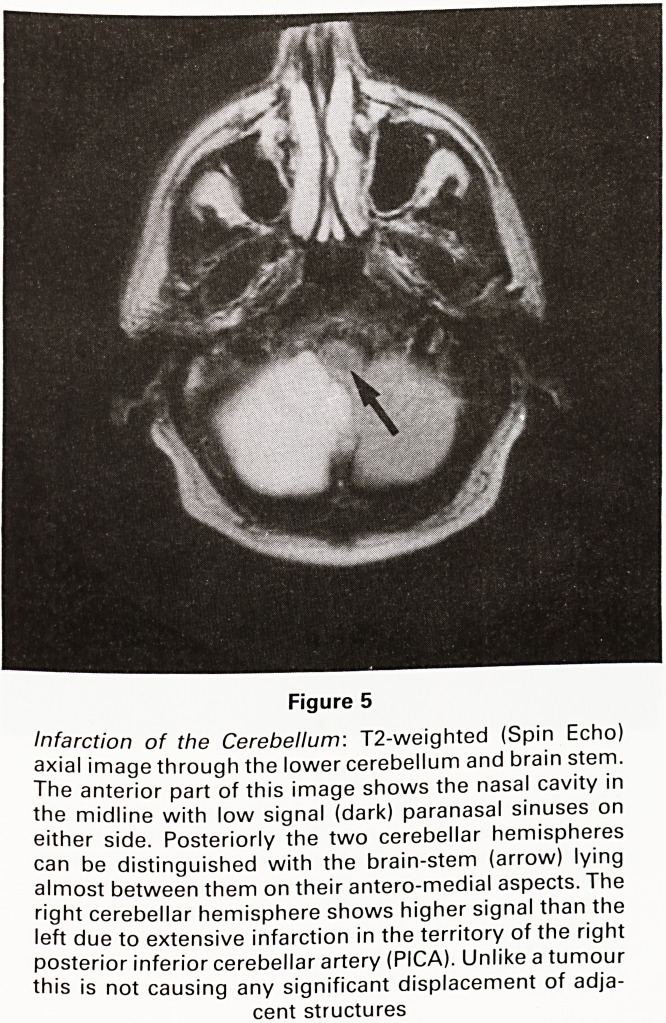


**Figure 6 f6:**
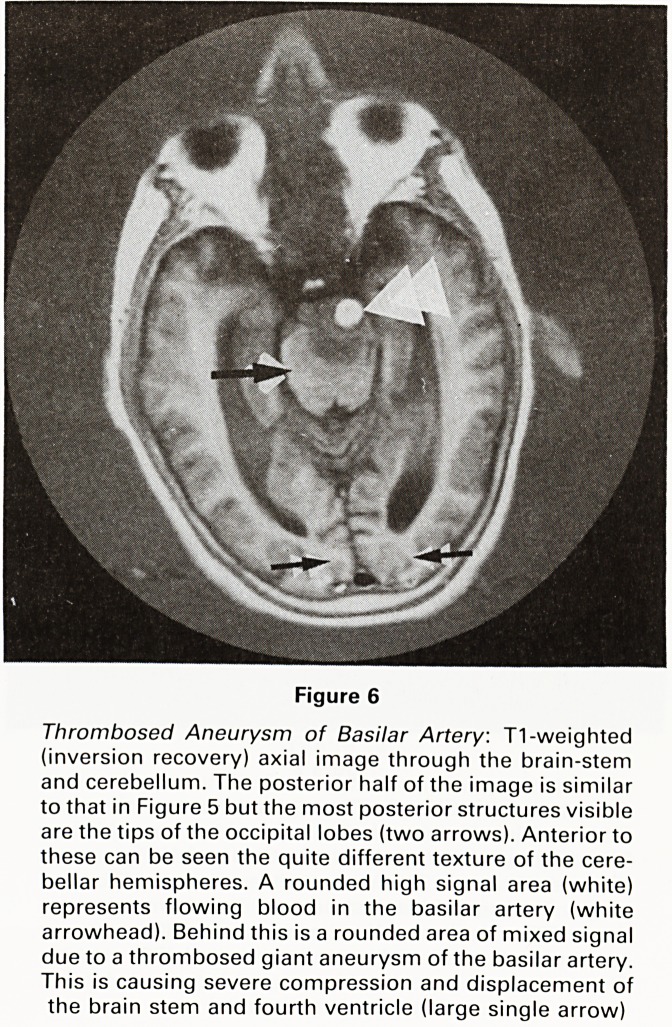


**Figure 7 f7:**
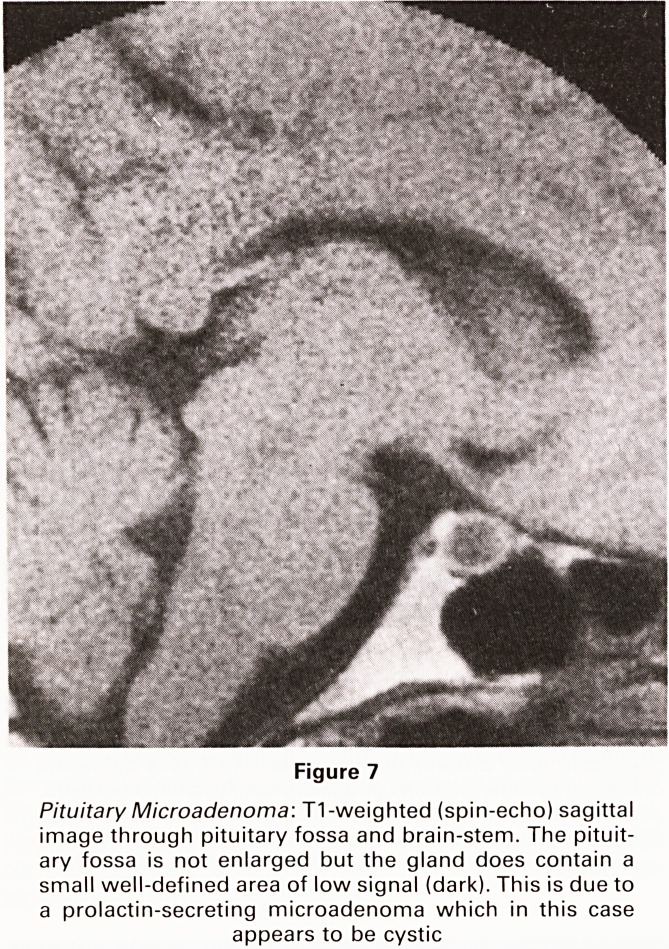


**Figure 8 f8:**
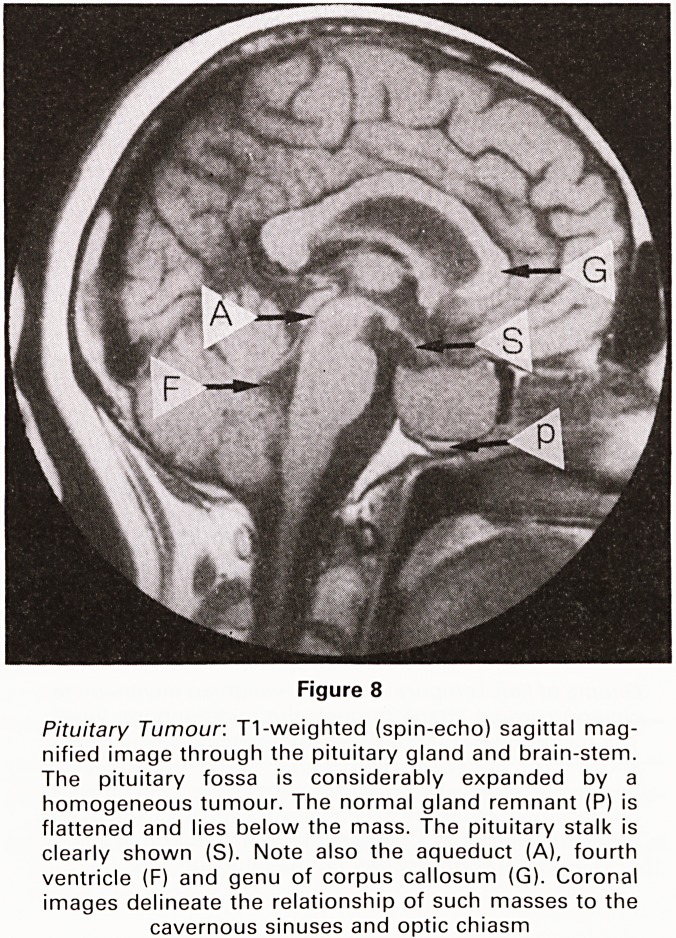


**Figure 9 f9:**
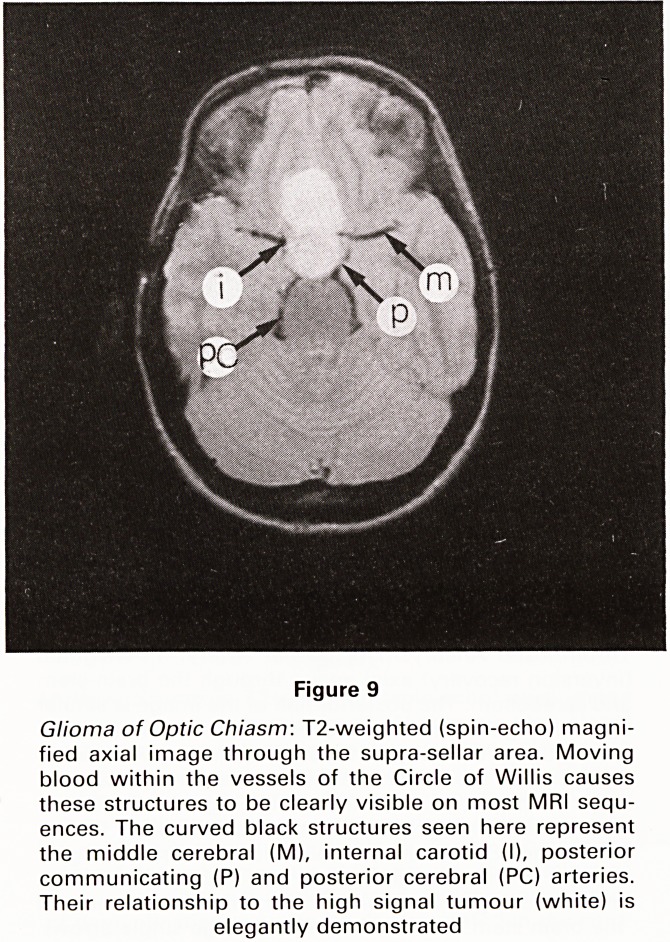


**Figure 10 f10:**
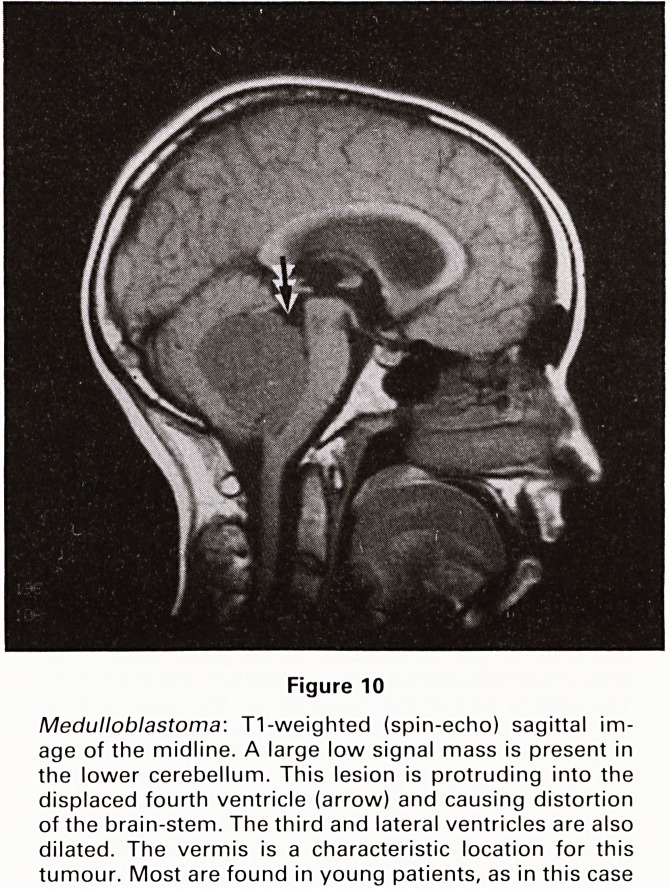


**Figure 11 f11:**
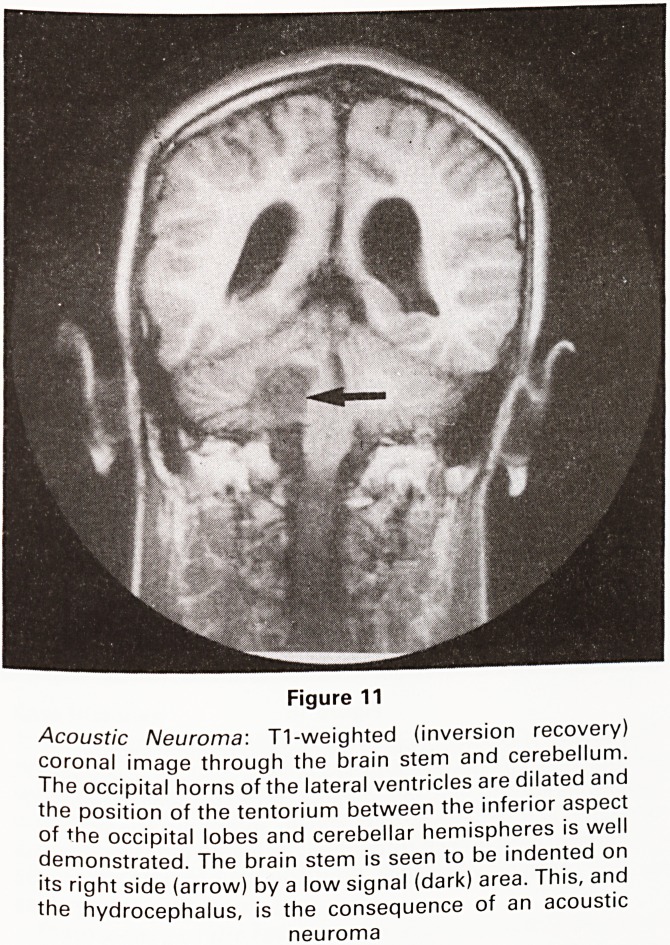


**Figure 12 f12:**
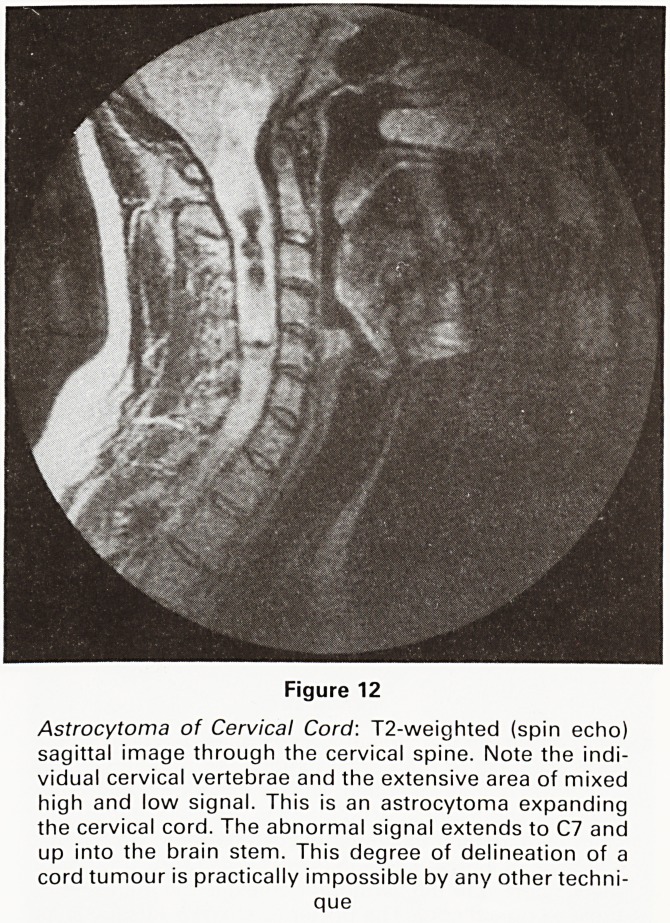


**Figure 13 f13:**
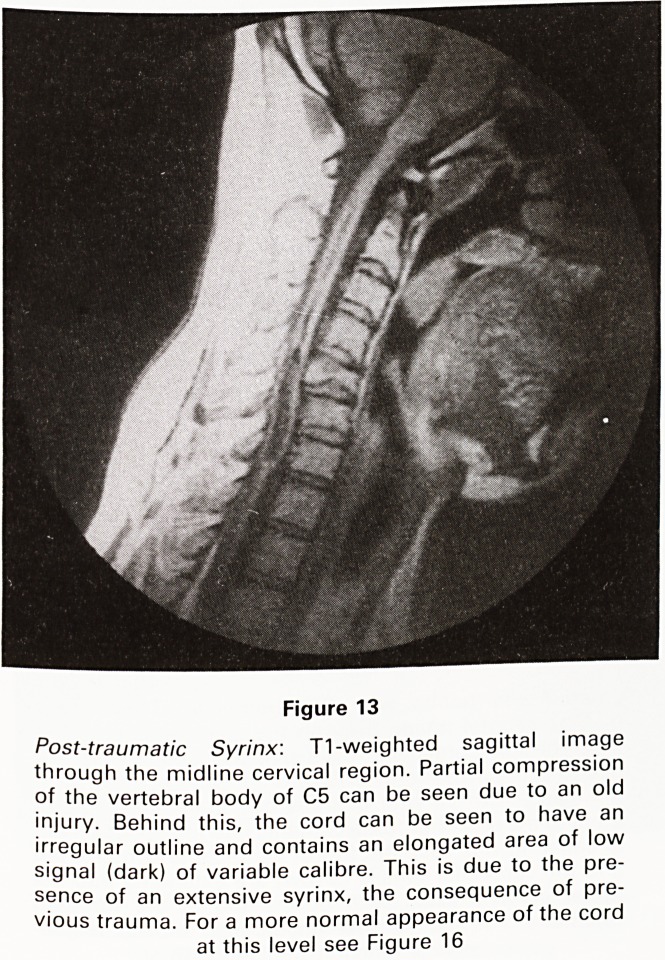


**Figure 14 f14:**
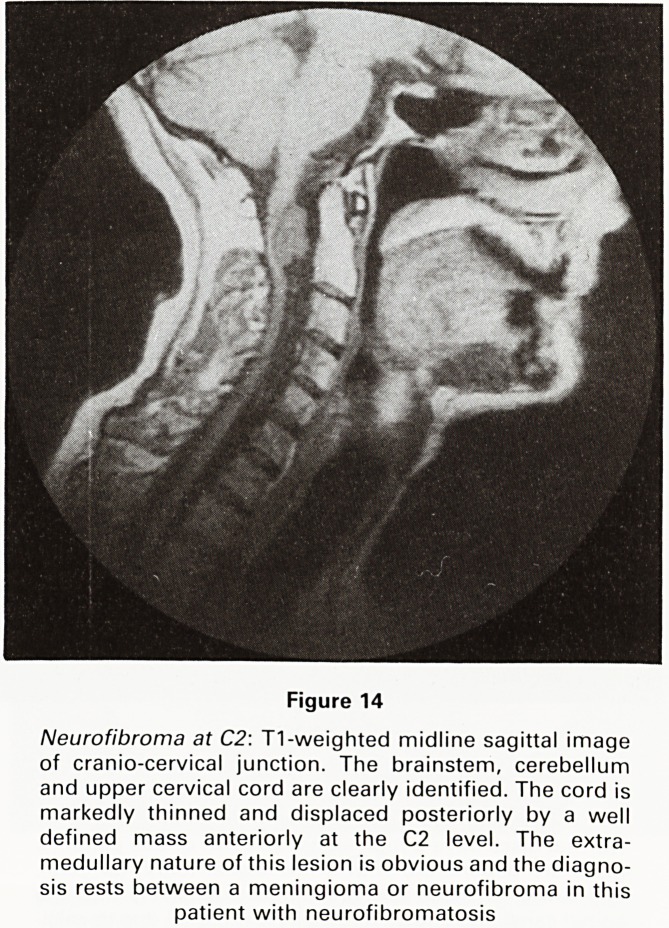


**Figure 15 f15:**
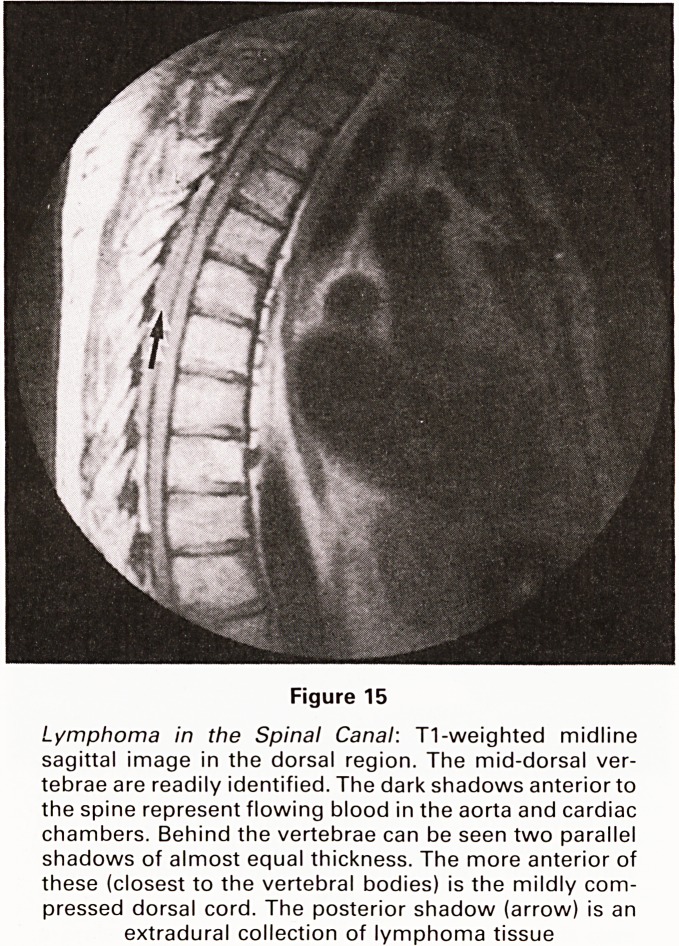


**Figure 16 f16:**
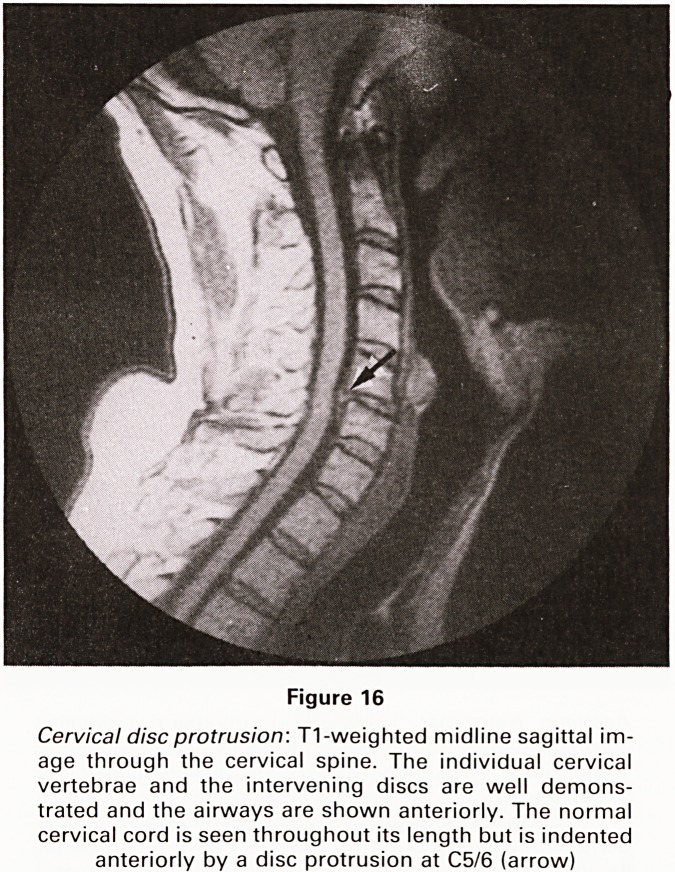


**Figure 17 f17:**
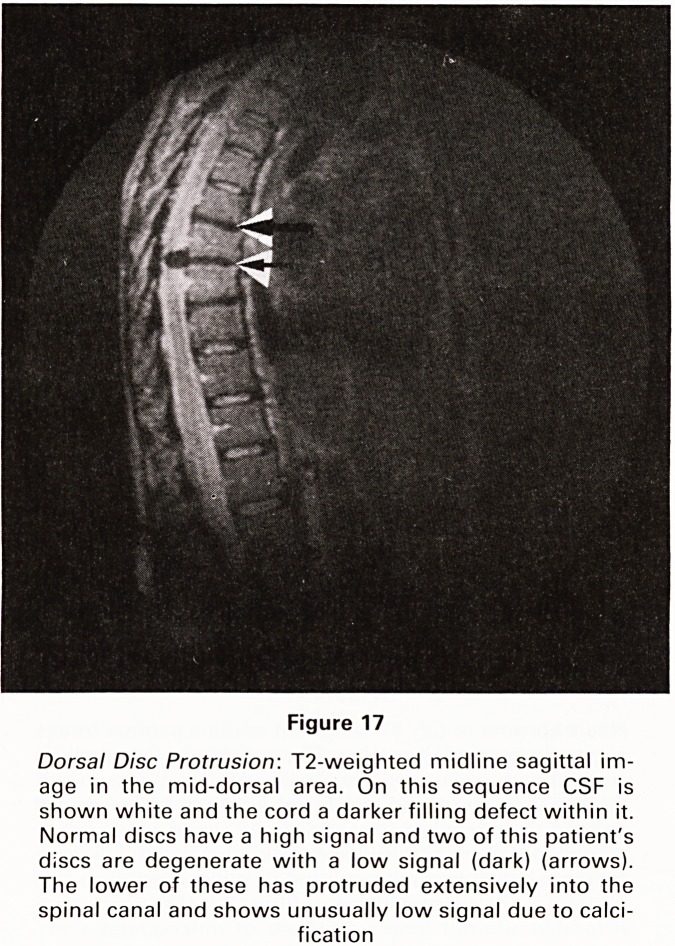


**Figure 18 f18:**